# Population modelling and genetics of a critically endangered Madagascan palm *Tahina spectabilis*

**DOI:** 10.1002/ece3.6137

**Published:** 2020-03-02

**Authors:** Alison Shapcott, Heather James, Laura Simmons, Yoko Shimizu, Lauren Gardiner, David Rabehevitra, Rokiman Letsara, Stuart Cable, John Dransfield, William J. Baker, Mijoro Rakotoarinivo

**Affiliations:** ^1^ Genecology Research Centre School of Science and Engineering University of the Sunshine Coast Maroochydore Qld Australia; ^2^ Cambridge University Herbarium Department of Plant Sciences University of Cambridge Cambridge UK; ^3^ Kew Madagascar Conservation Centre Ivandry Madagascar; ^4^ Parc Botanique et Zoologique deTsimbazaza Antananarivo Madagascar; ^5^ Royal Botanic Gardens, Kew Richmond UK; ^6^ Département de Biologie et Ecologie Végétales Faculté des Sciences Université d'Antananarivo Antananarivo Madagascar

**Keywords:** conservation genetics, demographic population growth modelling, population viability analysis, species distribution modelling

## Abstract

Madagascar is home to 208 indigenous palm species, almost all of them endemic and >80% of which are endangered. We undertook complete population census and sampling for genetic analysis of a relatively recently discovered giant fan palm, the Critically Endangered *Tahina spectablis* in 2008 and 2016. Our 2016 study included newly discovered populations and added to our genetic study. We incorporated these new populations into species distribution niche model (SDM) and projected these onto maps of the region. We developed population matrix models based on observed demographic data to model population change and predict the species vulnerability to extinction by undertaking population viability analysis (PVA). We investigated the potential conservation value of reintroduced planted populations within the species potential suitable habitat. We found that the population studied in 2008 had grown in size due to seedling regeneration but had declined in the number of reproductively mature plants, and we were able to estimate that the species reproduces and dies after approximately 70 years. Our models suggest that if the habitat where it resides continues to be protected the species is unlikely to go extinct due to inherent population decline and that it will likely experience significant population growth after approximately 80 years due to the reproductive and life cycle attributes of the species. The newly discovered populations contain more genetic diversity than the first discovered southern population which is genetically depauperate. The species appears to demonstrate a pattern of dispersal leading to isolated founder plants which may eventually lead to population development depending on local establishment opportunities. The conservation efforts currently put in place including the reintroduction of plants within the species potential suitable habitat if maintained are thought likely to enable the species to sustain itself but it remains vulnerable to anthropogenic impacts.

## Abstract 2

We studied the genetics, population ecology and suitable habitat for *Tahina spectabilis* a critically endangered palm from Madagascar to assess its long term viability. We found that current conservation measures may enable the species to persist. Its endangerment is not due to intrinsic population decline.

## 1 INTRODUCTION

There have been a startling number of plant species extinctions recorded in recent times, but equally, the number of species discovered and rediscovered indicates more field studies are needed to document what we have and where it grows (Humphreys, Govaerts, Fininski, Lughadha, & Vorontsova, 2019). Madagascar is one of the countries identified as having lost a disproportionately high number of plant species in modern times (Humphreys et al., 2019). It is one of the world's most threatened biodiversity hotspots with over 80 percent of its flora being endemic, coupled with widespread habitat degradation (Gautier & Goodman, 2003; Meyers, Mittermeier, Mittermeier, Fonseca, & Kents, 2000; Rakotoarinivo, Dransfield, Bachman, Moat, & Baker, 2014). Palms have been identified as approximately four times more at risk of extinction than other plant groups in Madagascar (Rakotoarinivo et al., 2014). A total of 41 species of endemic palms have been discovered since 1995 in Madagascar (Govaerts, Dransfield, Zona, Hodel, & Henderson, 2019), one of the most significant being the Critically Endangered *Tahina spectabilis* (tribe Chuniophoeniceae of subfamily Coryphoideae), due to its phylogenetic distinctiveness within the Madagascan palm flora (Baker et al., 2009; Dransfield & Rakotoarinivo, 2011; Dransfield, Uhl, et al., 2008; Rakotoarinivo et al., 2014). While the protected areas encompass many species, those with highly restricted distributions such as *T. spectabilis* are frequently missed (Heywood, 2019; Rakotoarinivo et al., 2014).

Endangered species recovery programs for plants now increasingly add translocations and reintroductions to secure species survival as well as in situ and ex situ conservation approaches, particularly where populations are critically small, restricted, and unprotected (Godefroid et al., 2011; Heywood, 2019; Silcock et al., 2019; Weeks et al., 2011). Success of translocations for reintroduction of threatened species will depend on knowledge of species' habitat distribution, biology and ecology, genetic diversity, and population dynamics (Godefroid et al., 2011; Heywood, 2019; Silcock et al., 2019; Weeks et al., 2011). *Tahina spectabilis*, while only recently discovered and named in 2008, was quickly secured with ex situ conservation measures which involved seed harvesting and international distribution (Dransfield, Rakotoarinivo, et al., 2008; Gardiner, Rabehevitra, Letsara, & Shapcott, 2017). This regulated local collection of seed also resulted in the opportunistic planting of specimens within the expected suitable habitat of the species (Dransfield, Rakotoarinivo, et al., 2008) close to the original population location (Gardiner, Rabehevitra, Letsara, et al., 2017). The recent discovery of a second population location to the north of the known and originally predicted habitat has led to the need to revise the original species habitat distribution model as well as to reassess the species extent of occurrence (EOO and area of occupancy (AOO) for IUCN conservation assessments (Dransfield, Rakotoarinivo, et al., 2008; Gardiner, Rabehevitra, Letsara, et al., 2017; Rakotoarinivo & Dransfield, 2012).

*Tahina specatabilis* is a hapaxanthic species, producing a massive terminal inflorescence that can give rise to many thousands of pale green fruits approximately 3 cm long and 2 cm in diameter before the plant subsequently dies (Dransfield, Rakotoarinivo, et al., 2008). The many thousands of small flowers on the inflorescence attract large numbers of insects (X. Metz, pers. obs.) that could be potential pollinators. Seeds could be dispersed by lemurs, bats, or parrots, which have been observed in the area (A. Shapcott, pers. obs.). Given this life cycle, small populations of adults can potentially be lost if plants reproduce too infrequently or if the resulting seed crop is aborted or destroyed by predation as the species depends of successful regeneration of seedlings. Palms play a particularly important role in human lives in poorer countries, such as Madagascar; however, they are often destructively harvested, for example, for palm heart consumption or construction materials, and in the case of *T. spectabilis*, that would lead to loss of reproductive potential as well as adult population size (Gruca, Blanch‐Overgaard, Dransfield, & Balslev, 2016; Melito, Faria, Amorim, & Cazetta, 2014; Rakotoarinivo et al., 2014; Shapcott, Quinn, Rakotoarinivo, & Dransfield, 2012).

The area in which *T. spectabilis* is found is known for its strongly seasonal climate that can support relatively few palm species compared to the species‐rich eastern rainforests of Madagascar (Dransfield & Rakotoarinivo, 2011; Dransfield, Rakotoarinivo, et al., 2008; Jury, 2003). The vegetation is savannah interspersed with seasonally dry deciduous western forest often associated with tertiary karst limestone outcrops (tsingy); small scale cattle grazing is common in the area, and due to a combination of local politics and agricultural practices, landscape level fires are common (Jury, 2003; Kull, 2002). While many palm species which occupy a savannah landscape are thought to be fire resistant, the combination of changed fire regimes and cattle grazing has been shown to impact the demographic growth of populations of some species (Abrahamson, 1999; Arneaud, Farrell, & Oatham, 2017; Mandle & Ticktin, 2012). Some local measures have been put in place at the original locations first visited in 2008 to protect *T. spectabilis* from fire and cattle trampling, considered to be threats to this species in situ (Gardiner, Rabehevitra, Letsara, et al., 2017; Gardiner, Rabehevitra, & Rajaonilaza, 2017). Demographic population modelling approaches have been widely used in palm studies to predict the impacts of differing threats on species including fire, grazing and harvesting and have proven a useful tool for conservation management (Arneaud et al., 2017; Gamba‐Trimiño, Bernal, & Bittner, 2011; Mandle & Ticktin, 2012; Martínez‐Ballesté, Martorell, Martínez‐Ramos, & Caballero, 2005; Melito et al., 2014; Navarro, Galeano, & Bernal, 2011; Pinéro, Martinez‐Ramos, & Sarukhan, 1984). The application of these approaches to investigate *T. spectabilis* population growth is likely to provide insights into its long‐term viability.

While much has been learnt in the past decades, relatively little is known about the ecology or genetics of Madagascan palm species (Dransfield & Beentje, 1995; Gardiner, Rakotoarinivo, Rajaovelona, & Clubbe, 2017; Ratsirarson, Silander, & Richard, 1996; Shapcott et al., 2007, 2012). Small population sizes can lead to reduced genetic diversity due to inbreeding, random drift, and reduced gene flow (Hooftman & Diemer, 2002; Leimu, Mutiikainen, Koricheva, & Fischer, 2006). Effective conservation and restoration of rare species uses information such as genetic diversity and interpopulation genetic relationships (Byrne, Stone, & Millar, 2011; Godefroid et al., 2011; Shapcott, Lamont, Conroy, James, & Shimizu‐Kimura, 2017; Weeks et al., 2011). Geographic projections of species distribution models (SDM) have been used to assist with interpreting the genetic patterns in endangered species as well as targeting areas for population searches and reintroductions (Pfab & Witkowski, 1997; Shapcott et al., 2012, 2017; Shapcott & Powell, 2011; Thode et al., 2014).

This study surveyed *T. spectabilis* populations in 2008 and again in 2016 to assess the demographic structure, population size, genetic diversity, and relationships, across the known species distribution in Madagascar. The viability and threats to the species were assessed with the assistance of population demographic matrix modelling PVA, and the impacts of the expanded known distribution since discovery were investigated. Specifically, we asked if the species populations were increasing, stable, or decreasing and what variables are most likely to impact on its long‐term viability? Are the newly discovered populations genetically distinct from the original known population and what do the patterns of genetic diversity tell us about the species at a landscape level? In addition, we asked what contribution are the locally planted individuals likely to have for the conservation of this species and how long is it likely to take before these or other isolated individuals contribute to develop new populations?

## 2 METHODS

All known populations of *T. spectabilis* were visited in 2008, and the coordinates of their locations recorded with a GPS. The relative locations of all individuals were systematically mapped as *X* *Y* coordinates along contiguous belt transects (20 m × 30 m) encompassing the entire population. The GPS location was taken at the start and finish of each transect as well as the compass direction. These data were later used to construct georeferenced maps of the relative locations of each plant by using trigonometry to adjust for variable transect direction. For each plant, the following data were recorded: height of the trunk to the base of the lowest leaf sheath or if no trunk, the total height of the plant; diameter or number of fronds if larger than seedling and lacking a trunk. The locations of three adult plants that had flowered and died in previous years (pre 2006, 2006, and 2007) were also recorded. Samples from mature leaves were collected from every plant above 15 cm height except one for genetic analysis. Samples were collected from a subsample of seedlings <15 cm. Samples were surface cleaned with paper towel, cut into smaller pieces, and stored in individually labelled sealed plastic bags with silica gel. In addition, silica gel‐dried leaf material from 25 seedlings propagated at RBG Kew from seed collected from the 2006 mother tree was also obtained to be included in the genetic analysis.

The original three populations were relocated in 2016 and resurveyed using the same systematic belt‐transect methods as used previously (Gardiner, Rabehevitra, Letsara, et al., 2017). However, to enable ongoing population recording by the local village personnel, we classified the plants into simple size classes (estimated using body dimensions) as follows where: Seedling 1 (S1): seedlings up to 30 cm; S2: plants between 30 cm and 1 m; Juveniles 1 (J1): plants with no trunk height between 1 and 2 m to the top of the leaves; J2: plants with no trunk and height >2 m to the top of the leaves; Adult 1(A1):plants with a trunk up to 2 m to base of leaf sheath; A2: plants with a trunk >2 but <6 m to the base of leaf sheath; and A3: plants with a trunk 6 m or taller to base of the leaf sheath. These were then retrospectively applied to the 2008 survey data. In the time since 2008, local surveys (X. Metz, unpub. data) had recorded the presence and location of reproducing trees. The locations of previously recorded trees (2008 survey) were used to compare with the 2016 survey and maps sent by X. Metz to identify and locate which trees had reproduced and to match the plants between surveys. The dates of all known reproductive events were matched with the dates for ENSO and ISO events to see if these may explain the timing of reproductive events.

**Figure 1:**
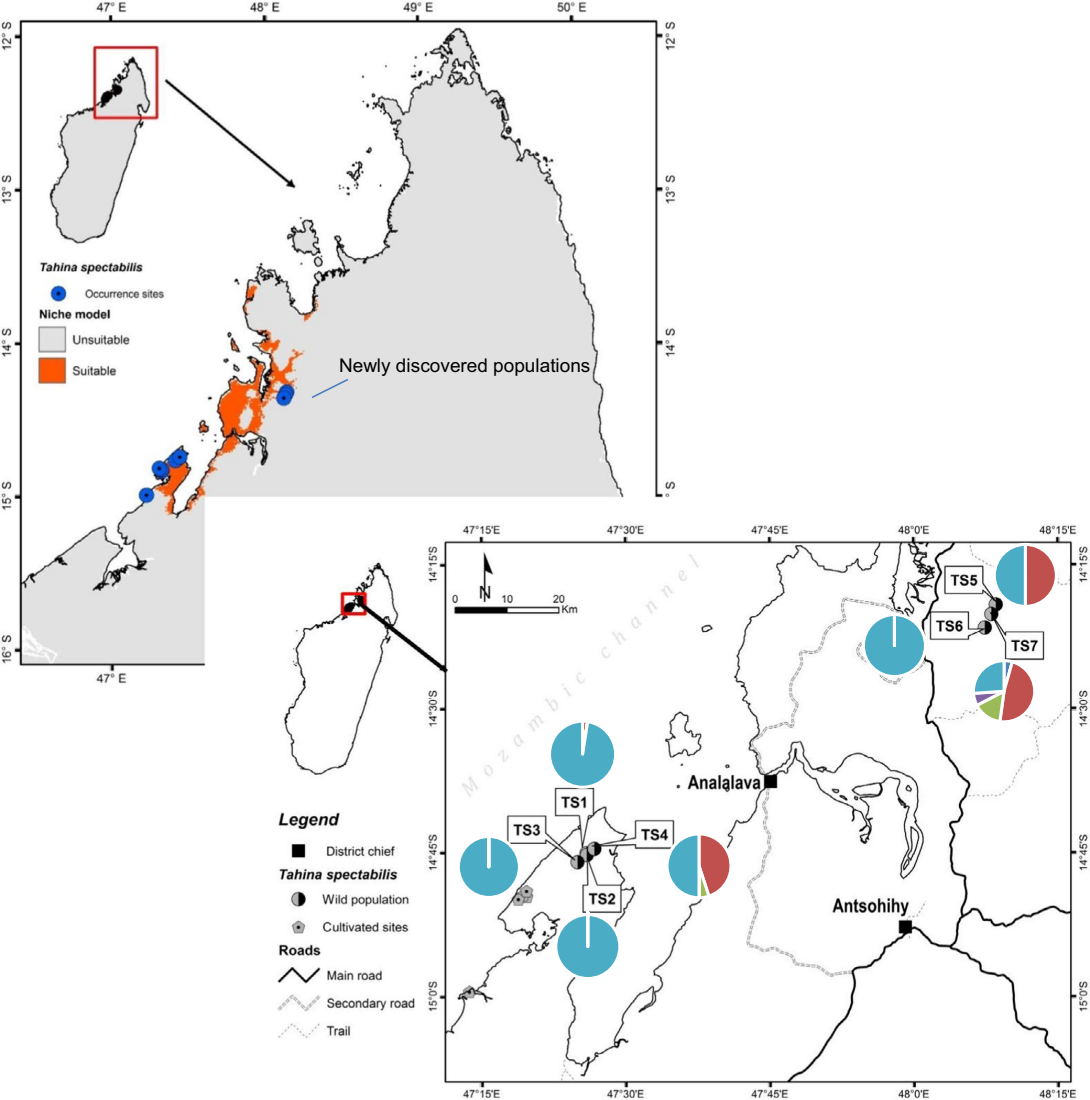
Map of Madagascar showing the *Tahina spectablis* locations and predicted suitable habitat as well as allelic frequency pie charts for the locus Aacu 7which highlight genetic composition similarities. The locations of the cultivated (planted) populations are also indicated

As a result of local searches, additional populations (TS4–TS7; Figure 1) were also located (Gardiner, Rabehevitra, Letsara, et al., 2017), and these entire populations systematically surveyed as described above. At the new locations a sample of leaf material was taken and stored in silica gel from each plant larger than 30 cm or a subset of small plants. These were used in subsequent genetic analysis to compare with the original 2008 samples.

In addition to wild populations, *T. spectabilis* plants had been propagated from the seed harvested in 2006/7/8/11 and planted at various locations thought to be within its natural predicted range based on the suitable habitat modelling undertaken by Dransfield, Rakotoarinivo, et al. (2008). The surviving cultivated plants were also surveyed during the 2016 census and their sizes and locations recorded. In 2019, they were recounted by local land managers at the respective sites and the numbers of plants established at each cultivated site as of 2019 documented (C. de Foucault, A. Andrianandraina, personal communication).

### 2.1 Habitat extent and modelling

The geographic coordinates of each site location was input to Google Earth Pro (http://www.earth.google.com), and the geographic distances between the populations were then determined using the ruler function The area occupied by each population was determined either directly from transect data or by constructing perimeter polygons in Google Earth Pro and using the area calculator. This enabled the expansion of the area of the TS1 population from 2008 to 2016 to be determined. The estimations of extent of occurrence (EOO) and area of occupancy (AOO) were made using the original 2008, new 2016 as well as planted populations as of 2019 with the GeoCat Geospatial Conservation Assessment Tool (http://www.geocat.kew.org; IUCN, 2014).

In order to refine the potential distribution of *T. spectabilis*, a species distribution model was created with Maxent 2.2 using the current known occurrence sites and 24 ecological variables including 19 climatic parameters (Hijmans, Cameron, Parra, Jones, & Jarvis, 2005) such as temperature, precipitation and five layers related to habitat characteristics: elevation, slope (USGS, 2015), geology (Besairie, 1964), vegetation map (Moat & Smith, 2007), and the vegetation cover fraction (Jiang et al., 2006).

As a statistical program for species distribution modelling, Maxent calculates the probability of occurrence (from 0 to 100) of one species in each pixel of 30 m × 30 m resolution throughout the study area. At the same time, it identifies the most important factors governing the distribution of one taxon by giving a training gain for each variable selected; one factor is considered as important if the species displays a strong sensibility in relation to it, that is, a high value of the test gain (Phillips, Anderson, & Schapire, 2006). The variables having the most influence in the model were then hypothesized to be the most significant for the distribution of the palm. In order to provide good distribution models, SDM was carried out in two steps: firstly, a model involving all of the 24 environmental layers, and secondly, a model based on the 10 first responsive layers from the first operation (Table 1). A distribution model is considered to be better than the other based on the higher value of the Area Under Curve (AUC). Once the model is defined, a binary map was created based on the model provided by Maxent. The threshold of Minimum training presence was hence used to classify the pixel values into two categories: unsuitable sites for the species (new pixel value = 0) and suitable sites for the species.

**Table 1:** List of environmental layers utilized for providing predictive distribution model of the geographic range of *Tahina spectabilis* using Maxent software

	Model 01 (all layers)	Model 02 (10 selected layers)
BIO1 = Annual Mean Temperature	Yes	Yes
BIO2 = Mean Diurnal Range (Mean of monthly (max temp − min temp))	Yes	Yes
BIO3 = Isothermality (BIO2/BIO7) (× 100)	Yes	
BIO4 = Temperature Seasonality (standard deviation × 100)	Yes	Yes
BIO5 = Max Temperature of Warmest Month	Yes	
BIO6 = Min Temperature of Coldest Month	Yes	
BIO7 = Temperature Annual Range (BIO5–BIO6)	Yes	
BIO8 = Mean Temperature of Wettest Quarter	Yes	
BIO9 = Mean Temperature of Driest Quarter	Yes	
BIO10 = Mean Temperature of Warmest Quarter	Yes	
BIO11 = Mean Temperature of Coldest Quarter	Yes	
BIO12 = Annual Precipitation	Yes	Yes
BIO13 = Precipitation of Wettest Month	Yes	Yes
BIO14 = Precipitation of Driest Month	Yes	
BIO15 = Precipitation Seasonality (Coefficient of Variation)	Yes	
BIO16 = Precipitation of Wettest Quarter	Yes	
BIO17 = Precipitation of Driest Quarter	Yes	
BIO18 = Precipitation of Warmest Quarter	Yes	
BIO19 = Precipitation of Coldest Quarter	Yes	
Digital Elevation Model	Yes	Yes
Slope	Yes	Yes
Geological map (1/100,000)	Yes	Yes
Atlas of the vegetation of Madagascar	Yes	Yes
Vegetation cover fraction	Yes	Yes

### 2.2 Demographic modelling

The raw frequencies in each developmental stage were transformed to percentage of the population in each size category and plotted to investigate the shape of the population structure. The plants at TS1 were classified according to the seven developmental stages described above, and this field data were used to quantify the parameters and develop a life history model for *T. spectabilis* following similar methods to Simmons, Auld, Hutton, Baker, and Shapcott (2012). We used the detailed analysis of the TS1 population structure and relative locations and sizes of individuals in 2008 and 2016 to determine estimates of the numbers of individuals remaining in the same size category and those that transitioned to the next developmental stage in the eight year time step. Thus, calculations of survivorship (stage specific survivorship *Ss_i_*) and transition probabilities (stage‐transition survivorship *S_i_*) between development stages based on methods of Akçakaya, Burgman, and Ginzbur (1999), Gamba‐Trimiño et al. (2011) and Simmons et al. (2012).

The time spent in each stage class was estimated based on changes in abundance of plants over the 8 year time frame and the ability to track individual plants growth between the two time periods due to our detailed mapping of plants. This was used to estimate the mean residential time in each stage class (MRT) which allowed the mean life span (Mlf) of the species to be estimated (Akçakaya, 2005; Akçakaya et al., 1999). Note that standard deviations for these estimates are also calculated. Fecundity was zero for all stages except A3 (F_A3S1_) and was given as the estimated average number of seedlings produced per reproductive tree based on field data. As these data are based on seedling numbers per reproductive tree after seed harvesting has taken place, all models incorporate seed harvesting. We used the number of known trees that did or did not fruit successfully over the total time period since record keeping began for this species, to estimate the average frequency of reproduction.

A baseline Lefkovitch stage matrix was developed based on the parameters determined from the life history model developed above from field observations and input into RAMAS GIS v5.0 (Akçakaya, 2005). RAMAS GIS v5.0 (Akçakaya, 2005) was used to run a single population model to explore population growth parameters. To increase realism in the model, the time step was set to eight years, replications were set to 1,000, density dependence was set to be ceiling (also ran exponential), and stage specific to S1, S2, J1, J2 developmental stages affecting survival rates to simulate self‐thinning of high density seedling populations. The eight year time step was used to ensure our model parameters were consistent with the field observations. The maximum carrying capacity (Kmax) was set to approximately 10 times the TS1 population size (5,000) to allow for maximum capacity of seedling production and probably overestimates the potential for the habitat to accommodate this species. Maximum growth rate (*λ*) was set at 1.5 based on the observed overall population growth rate of 1.47 over the eight year time period at TS1. A 10 percent standard deviation in the stage matrix (also tried 20% *SD*) was used to account to sampling error in calculations and is the program default.

Environmental stochasticity was set to normal in the model developed within RAMAS GIS v5.0 (Akçakaya, 2005). Relative survival was set to 0.8 (but other options were tested), which reflects the very high survival rate observed in the field but allowed for some mortality. Relative fecundity was set at 0.2 based on the average frequency of reproductive events over the time period of record keeping (other frequencies were also trialed to see their impact). We used the population size and structure as of 2016 at TS1 as our starting population. Within the RAMAS demographic model, we trialed a variety of scenarios including catastrophe 1 where plants reproduce but do not produce seedlings in this case fec = 0 and the frequency of events based on observed field data which was converted to a probability. Thus as 2/7 observed reproductive events resulted in seed set failure the probability of seed set failure was initially set at a 0.2857. We also modelled a second catastrophe 2 to mimic potential scenarios of fire events killing seedlings and juveniles, plants with trunks are expected to survive fire based on observations and previous studies of other similar species (Mandle & Ticktin, 2012), and we modelled a combined catastrophe 1 and 2 scenario. These were initially modelled for 10 time steps (80 years). The probability of fire events were based initially on a fire occurring once in every 10 years, and the impacts of changed frequency was explored within the models as was the impact on survivorship of different stages. The model was run many times to explore the impact of variations on some of the parameters on the TS1 population growth.

After exploring single population models on TS1, we added all known populations to a metapopulation model that included spatial locations and initial population sizes and structure as of 2016 and we used the TS1 matrix model that included both fire and reproductive failure. Maximum dispersal distances were estimated based on nearest neighbor distances of wild populations. The estimates of maximum dispersal rates were based on the expected approximate age of single plants that are located adjacent to larger populations (one successful event per estimated age of the plant per distance from closest population), and average dispersal distances and rates are based on the observation of within population dispersal/expansion at TS1. This was then modelled for 25 time steps (200 years) and 15 time steps (120 years).The model trajectories including population growth, final stage structure, and extinction probabilities and trajectories were plotted in RAMAS GIS v5.0 (Akçakaya, 2005). These were conservative stochastic models based on real data the use of the longer time step that matched the time step of the data collected makes the models input data more robust for chance annual fluctuations.

### 2.3 Genetic analysis

All samples collected during the 2008 survey were used in the initial genetic study (James, 2010). Samples collected during the 2016 survey were subsequently analyzed. Approximately, 30–50 mg of silica dried plant tissue samples were frozen using liquid nitrogen and ground using a Retsch MM200 Tissue Lyser grinding mill (Qiagen) for 30 s at 23,000 rpm this was repeated several times. DNA was extracted and purified using the DNeasy Plant Mini‐kit (QIAGEN) as per Shapcott et al. (2012). Several samples of DNA from the original extractions representative of TS1, TS1 seedlings, TS2, and TS3 were added to the 2016 analysis to enable calibration and cross referencing.

Thirty‐eight microsatellite primers previously developed for other palm species were trialed for successful amplification of *T. spectabilis* for the initial genetic study using a subset of samples (James, 2010) as per Shapcott et al. (2012). Of those that successfully amplified product within the expected size range further standard optimization of PCR conditions were trialed as needed such as altering MgCl_2_ the number of PCR cycles and testing for optimal annealing temperature to obtain repeatable clean product. Following this process, nine primers that gave optimal results within the expected size range were used for genetic analysis. These were; Pd15, Pd32, Pd50, Pd57 (*Phoenix dactylifera*; Billotte et al., 2004); Eg254, Eg391, Eg476 (*Elaeis guineensis*; Billotte et al., 2001); Ob11 (*Oenocarpus bataua* Montufar, Mariac, Pham, & Pintaud, 2007), and Aacu07 (*Acrocomia aculeata*; Nucci et al., 2008). Only minor changes to published PCR conditions were made during the optimization of the primers. Each primer was fluorescently forward end labelled with NED, FAM or HEX for the 2008 samples (James, 2010 as per Shapcott et al., 2012) or with NED, FAM, PET or VIC for the 2016 samples to allow multiplexing as per Shapcott et al. (2017).

The PCR products underwent fragment analysis on an AB3500 Genetic Analyser (Applied Biosystems) to determine the genotype of each individual at each locus. GENEMAPPER v4.1 software (Applied Biosystems) was used to score fragments according to alleles and loci, double checked manually to ensure accuracy to expected banding size. The repeated samples from the 2008 collections that were run with the 2016 collection were used to confirm consistency of genetic scoring to alleles, and adjustments were made to bp size of alleles present due to the different machine runs between original 2008 and 2016 samples. Of the nine loci tested only four were polymorphic within the species Pd15, Ob11, Aacu07, Pd32 and these were used for further analysis. We acknowledge the limited resolution of so few variable loci and results need to viewed with caution.

Allelic frequencies were calculated at each locus for each site and were used to determine for each site: the mean number of alleles per locus (*A*), the percentage of polymorphic loci (*P*), mean expected heterozygosity (*H*_e_), mean observed heterozygosity (*H*_o_), and the fixation index (*F*) was also calculated using GenAlEx 6.4 (Peakall & Smouse, 2006). A Pairwise Popula A Pairwise Population Matrix of Nei's Genetic Distance was used to determine the level of relatedness among the populations and individuals, and a Principal Coordinates Analysis (PCoA) was undertaken using genetic distances between all individuals in the species using GenAlEx 6.4 (Peakall & Smouse, 2006). A UPGMA analyses were used to generate a dendrogram showing the genetic relationships among populations using Primer v 6 (Clarke & Gorley, 2001).

## 3 RESULTS

### 3.1 Demographics and habitat

Our survey in 2008 identified 394 wild growing *T. spectabilis* plants (78% seedlings;14% juveniles; 7% trunked) at three locations in close proximity (TS1, TS2, TS3), and further searches in 2016 documented 774 wild growing *T. spectabilis* plants (86% seedlings; 10% juveniles; 4% trunked) at a total of seven sites including the original three locations (Figure 1, Table 2). The majority of plants were found at TS1 (Table 2). In 2008, we counted 27 trees with trunks developed, 19 of which the trunks were 2 m or taller (A2, A3; Table 2). In 2016, we counted 27 trees with trunks, but only 14 of those had trunks 2 m or taller; five mature trunked trees had died after initiating flowering (Table 2). The population TS4, discovered in 2010, was composed entirely of 170 seedlings in 2016 (Figure 1; Table 2). Observations from the TS4 and TS1 populations suggest high seedling survivorship from S1 to S2 stages. The highly disturbed sites, TS5 and TS6, each consist of a single plant (Figure 1, Table 2). TS7 was located in a more intact vegetation patch nearby, consisting of 23 plants and occupying approx. 350 m^2^, but only has three trunked trees and only one with a trunk >2 m (Table 2).

**Table 2:** Demographic summary of *Tahina spectabilis* known wild (in situ) populations/sites at census in Sept 2008 and 2016

Site/Year	Size	Class							
	S1	S2	J1	J2	A1	A2	A3		
	<30 cm	<1 m	<2 m	>2 m	Trunk <2 m	trunk 2–5 m	Trunk 6 + m	Total	Area Ha
2008									
TS1	300	9	32	24	8	8	11	392	1.34
TS2	0	0	0	0	0	1	0	1	0.0004
TS3	0	0	0	0	1	0	0	1	0.0004
Total wild	300	9	32	24	9	9	11	394	1.35
2016									
TS1	108	382	28	32	13	8	6	577	2.76
TS2	0	0	0	0	0	0	1	1	0.0004
TS3	0	0	0	0	1	0	0	1	0.0004
TS4	33	137	0	0	0	0	0	170	0.005
TS5	0	0	0	0	1	0	0	1	0.0004
TS6	0	0	0	1	0	0	0	1	0.0004
TS7	0	5	6	9	2	1	0	23	0.035
Total wild	141	524	34	42	17	16	7	774	2.82

Note

Size classes are given where Juveniles 1 (J1): plant with no trunk but between 1 and 2 m to the top of leaves; J2: plant with no trunk but >2 m to the top of leaves; Adult 1 (A1): plant with trunk up to 2 m in height to base of leaf sheathes; A2: plant with trunk >2 m but <6 m in height to the base of leaf sheathes, and A3: plant with trunk 6 m or taller to base of leaf sheathes. The total number of plants year and area (Ha) of each population site occupied are given

The revised SDM indicates that the potential areas suitable for the species remain restricted to coastal areas in small parts of the northwest of Madagascar, between Analalava in the north and to the southern part of the Antsanifera peninsula (Figure 1). Of the two predicted models created, the one involving only 10 environmental layers gave higher AUC value, equal to 0.998 while AUC was 0.976 for the model where the whole 24 parameters were applied. According to this model (model 2; Table 1), the restriction of this species in the coastal plain and in this part of Madagascar could be explained mostly by the combination of temperature seasonality, the wettest month precipitation and the geological substrate. The three environmental parameters model up to 83% of the current distribution range according to the Maxent jackknife of factor importance. The predicted suitable area for *T. spectabilis* is homogeneously located on sandstones as geological substrate (percent contribution 12%) but climatically the species requires low temperature seasonality (contribution 48%), that is, where there is little variation in the mean monthly temperature (difference of 10–14°C between warmer and colder season) throughout the year and relatively high precipitation during the wettest month (February = 400–480 mm, out of about 1,500 mm/year; 23% important in the species modelling).

There is considerable potential suitable habitat within the northern region, particularly closer to the coast. The total Extent of Occurrence EOO of wild populations is 206,751 km^2^ and the AOO based on a 2 km grid is 24 km^2^; there is a considerable disjunction between the two known areas of occurrence (Table 3) although suitable areas are relatively predicted between the known occurrence sites (Figure 1).

**Table 3:** Summary of *Tahina spectabilis* population estimates

	Total plants	Total trunked	Total Trunked >6 m	EOO	AOO
2008 wild	394	29	11	0.359	12
2016 wild	774	33	6	206.751	24
2016 wild and planted	809	33	6	763.994	44

Note

The total number of plants counted at each census, the total number of trunked plants and the total number of plants with trunks 6 m and taller (IUCN adults) potentially capable of reproducing. Estimates of extent of occurrence (EOO) and Area of occupancy (AOO) in km^2^ are given, taking into account the contribution of planted populations of 2016.

**Figure 2:**
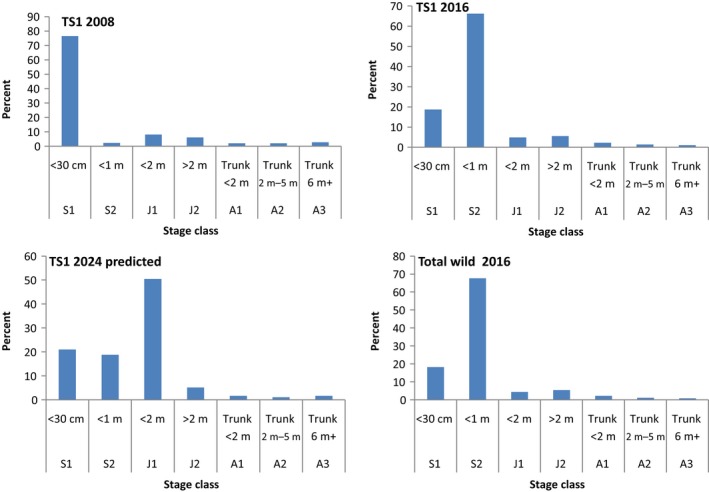
Demographic Size Structure of *Tahina spectabilis* TS1 population at 2008 and 2016 census and predicted for 2024 as well as the combined total size structure of the wild population across all known sites as of 2016

The TS1 population has grown overall between 2008 and 2016 despite five trunked adult plants reproducing and subsequently dying. The total number of trunked plants remained the same between the years due to a transition of five new plants to A1 (Table 2; Figure 2). There was evidence of local seed dispersal since 2008, as the extent of occupancy of the tsingy by *T. spectabilis* expanded from approximately 1.34 Ha in 2008 to 2.76 Ha in 2016 (Table 2), with plants now found around the entire circumference of the tsingy.

There was successful fruiting recorded in 2007/8, and the pulse of seedlings is evident in the elevated S1 size class (Figure 2). Five trees flowered/fruited between 2010 and 2011, and there was a report of an inflorescence developing in 2019 (A. Andrianandraina personal communication). Evidence of successful seedling recruitment from both the most recent and previous seed events is seen in the S1 and S2 size classes in 2016 (Table 2, Figure 2). Our predicted size distribution for 2,024 shows the maturing of the regeneration pulses observed in 2008 and 2016 (Figure 2). Trees that reproduced all have trunks 6 m or taller and reproduction can only happen once in the lifetime of each plant. Based on the combined seedling recruitment events and reproductive events, we estimated fecundity as measured by the average number of seedlings produced per reproductive adult was 120 (F_A3S1;_ Table 4).

**Table 4:** The estimates of Mean residence time in years (MRT) including the estimated standard deviation is given for each stage class for *Tahina spectabilis*

Stage	MRT (years)	Si	Ssi	Fec
S1	5 ± 2	S_S1S1_ = 0	S_S1S2_ = 0.8	0
S2	8 ± 2	S_S2S2_ = 0.01	S_S2J1_ = 0.9	0
J1	10 ± 3	S_J1J1_ = 0.59	S_J1J2_ = 0.4	0
J2	10 ± 4	S_J2J2_ = 0.79	S_J2A1_ = 0.2	0
A1	12 ± 4	S_A1A1_ = 0.4	S_A1A2_ = 0.6	0
A2	12 ± 4	S_A2A2_ = 0.4	S_A2A3_ = 0.6	0
A3	16 ± 5	S_A3A3_ = 0.4		120
Total	73 ± 24			

Note

The probability estimates of a plant surviving and staying in the same stage class (Si) or transitioning to the next stage class (Ssi) within the eight year time period are given for each stage class. The expected fecundity at each stage class (Fec) is also given as the number of seedling produced per reproductive plant.

There has been some fencing to reduce cattle trampling, and there was evidence of high survivorship of seedlings in areas where seedlings were protected at TS1, but a loss of seedlings was documented in exposed areas. The A1 and A2 stages had no observed mortality in the 8 years between surveys. It appears to take longer than 8 years for trees to grow from a trunk <2 m tall (A1) to 1 > 2 m tall (A2; Table 4). We found extreme variability among plants at TS1, TS2, and TS3 with high growth rates of some individual plants (Table 4). Based on the transition rate between the differing stages observed at the 8 year time interval, we estimate it takes approximately 73 years (±24 years) for trees to reproduce, but we expect a large variation in individual growth rates (Table 4).

**Figure 3:**
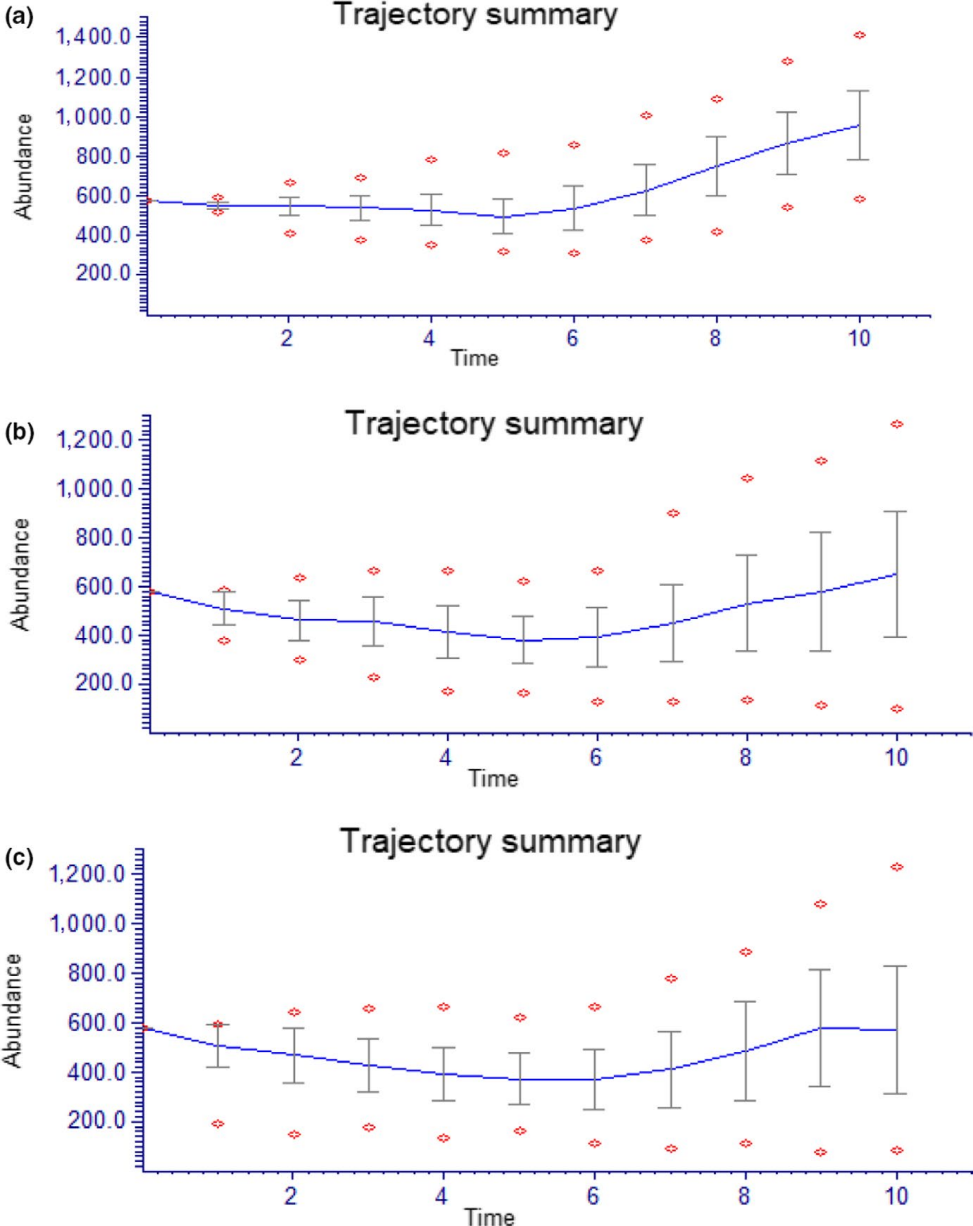
Population growth models the *Tahina spectabilis* TS1 population trajectory over 10 eight year time steps (a) no catastrophes (b) with seed failure (c) with both seed failure and fire catastrophes. The mean trajectory is shown with standard error bars and outliers indicated by diamonds

We trialed several variations of demographic parameters in initial growth models that were run for TS1 based on observed growth parameters, all resulted in population increases over the time frame modelled (Figure 3a). However, when models added potential impacts of failed seed set (Catastrophe 1) the population remained stable with little increase (Figure 3b). The model that included the chance of seedling failure due to fire (Catastrophe 2) combined with reproductive failure (Catastrophe 1), maintained the population at similar sizes to present (Figure 3c), and no models predicted extinction of this population.

**Figure 4:**
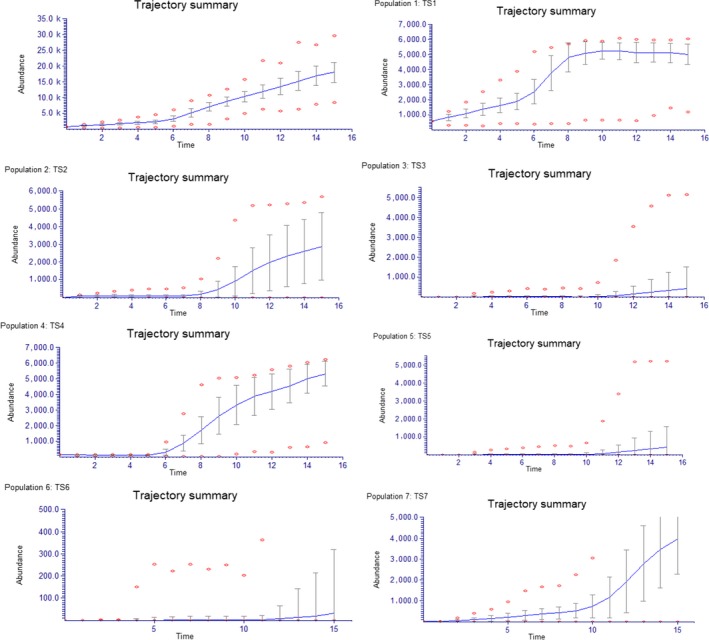
Trajectory summary for the whole spatial distribution of *Tahina spectabilis* as a metapopulation run over 15 eight year time steps, plus each individual populations are shown. Models were run with both seed failure and fire catastrophes and ceiling competition. (a) whole metpopulation; TS1; TS2; TS3; TS4; TS5; TS6:TS7

The average distance to nearest neighbor population is 1.9 km and the presence of single plants at several sites suggests successful seed dispersal within a 3 km range (Table 5). Our metapopulation model for *T. spectablilis* that includes all known wild plants, predicts the species overall population will slowly increase most noticeably after approximately 60 years (Figure 4). It is predicted to take at least 80 years for the sites with single plants to develop into multi‐aged populations due to the time to reproductive maturity, slightly sooner for TS4 as it is composed of established seedlings (Figure 4). Under these conditions and excluding other external factors, no models predict the extinction of *T. spectabilis* within the next 200 years. Sensitivity analysis indicated that the species population growth is most affected by the frequency of reproductive failure (Catastrophe 1).

**Table 5:** Summary of genetic diversity measures for each *Tahina spectabilis* population averaged across the four polymorphic loci studied

	*N*	*A*	*H*_e_	*H*_o_	*P*	*F*	NN
TS1	127	1.50	0.01	0.01	50	0.156	0.32
TS2	1	1.00	0.00	0.00	0	NA	0.32
TS3	1	1.00	0.00	0.00	0	NA	2.26
TS4	10	2.50	0.44	0.18	100	0.627	1.73
TS5	1	1.30	0.13	0.25	25	NA	2.10
TS6	1	1.00	0.00	0.00	0	NA	2.88
TS7	23	2.75	0.36	0.16	100	0.358	2.10
Mean		1.54	0.13	0.08	39	0.261	1.92

*N* = number of samples: *A* = mean number of alleles per locus; *H*_e_ = mean expected heterozygosity; *H*_o_ mean observed heterozygosity; *P* = percentage of loci polymorphic; *F* fixation index inbreeding coefficient Distance to nearest neighbor population NN (km).

In 2016, we documented well established cultivated plants, planted for conservation purposes at several locations (Figure 1). The sizes of these plants in 2016 were highly variable, ranging from <1 m tall to well over 2 m tall (S1, S2, J1 J2). In 2019, there are 22 plants confirmed to be established at four locations within the Verama Cashew plantation vicinity (Figure 1). Another site further south has another 10 plants established as of 2019 (Figure 1). Thus, there are an additional 32 nontrunked plants established at five “recovery/reintroduction” sites at the southern end of the *T. spectabilis* range. The combined wild and planted *T. spectabilis* populations, extends the EOO by 3.5 times to approx. 763,994 km^2^ and nearly doubles the AOO in size from 24 to 44 km^2^ (Table 3). We also have confirmed three *T. spectablis* plants have been successfully established at The Botanic Gardens of Majunga University, and there are also established plants at the Tsimbazaza Botanic Gardens in Antannarivo. Our modelling indicates that it will take these planted trees at least 50–70 years to reach reproductive maturity after which seedlings may expand the populations if suitable conditions exist (Table 4).

### 3.2 Genetics

**Figure 5:**
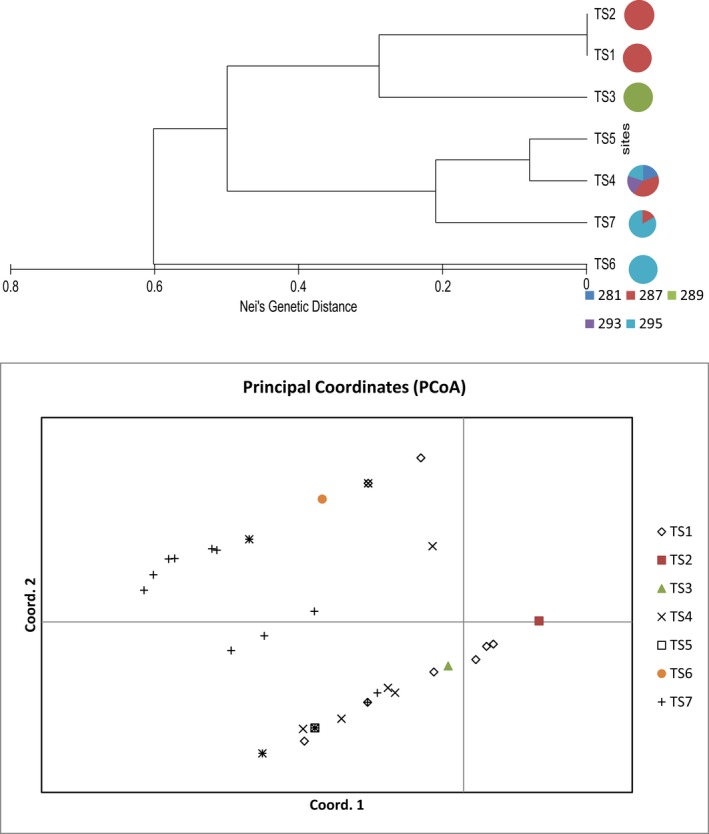
Top UPGMA dendrogram using Nei's genetic distance measure to show the relationship among the *Tahina spectabilis* sites. Pie charts beside the site codes represent the allelic proportions at the Pd32 locus nb missing data for TS5 This indicates the unique allele present at TS3. Bottom Principal coordinates analysis of *Tahina spectabilis* individuals analyzed for genetic variation across 4 polymorphic markers individuals are labelled according to the location at which they were sampled. Coordinate 1 accounts for 49.89 percent of the variation in the data while coordinate 2 accounts for 18.16 of the variation

Of the nine loci tested only four were polymorphic within the species Pd15, Ob11, Aacu07, Pd32 (Table 6) due to the low variability of loci the results need to be viewed with caution and we have limited our analysis based on the limitations of the data. The results indicate that the original known population TS1 contains low diversity compared with other smaller, newly discovered populations (Tables 5 and 6). The newly discovered populations possess two polymorphic loci that were monomorphic in the original TS1, TS2, and TS3 populations (Table 6). When only the polymorphic loci are considered in diversity measures, the seedling population TS4 is remarkably genetically diverse, as is TS7 (Table 5). Pd32 and Aacu07 were the most variable loci with 5 alleles recorded in the species and TS7 containing all alleles of Aacu07 (Table 6; Figures 1 and 5). All populations were inbred with allelic fixation index (F) > 0 (Table 5). TS1 is monophorphic at all but one locus, Aacu07, where a few adult individuals contain an alternative allele 156 (Table 6). This allele is also shared by TS4, TS5, and TS7 (Table 6; Figure 1). Our sample of seedlings from TS1 found that they arose from one individual, and this individual was of the few plants that was heterozygous for the only polymorphic allele found in this population, thus the seed distributed around the world sampled the limited genetic diversity found in this population.

**Table 6:** Allelic frequencies at each variable loci tested in *Tahina spectabilis* are given for each population sampled. Note TS2, 3,5,6 are single individuals

Loci/allele	Population						
	TS1	TS2	TS3	TS4	TS5	TS6	TS7
Pd15							
123	1.00	1.00	1.00	0.85	1.00	1.00	0.95
129	0.00	0.00	0.00	0.25	0.00	0.00	0.05
Ob11							
243	0.00	0.00	0.00	0.14	0.00	0.00	0.00
255	1.00	1.00	1.00	0.86	1.00	1.00	0.73
267	0.00	0.00	0.00	0.00	0.00	0.00	0.27
Aacu07							
154	0.00	0.00	0.00	0.00	0.00	0.00	0.04
156	0.03	0.00	0.00	0.45	0.50	0.00	0.48
158	0.00	0.00	0.00	0.05	0.00	0.00	0.15
166	0.00	0.00	0.00	0.00	0.00	0.00	0.06
170	0.98	1.00	1.00	0.50	0.50	1.00	0.26
Pd32							
281	0.00	0.00	0.00	0.20	NA	0.00	0.00
287	1.00	1.00	0.00	0.40	NA	0.00	0.17
289	0.00	0.00	1.00	0.00	NA	0.00	0.00
293	0.00	0.00	0.00	0.20	NA	0.00	0.00
295	0.00	0.00	0.00	0.20	NA	1.00	0.83

TS3 does not appear to have arisen from TS1 as TS3 is homozygous for a unique allele at locus Pd32, which is restricted to this population (Table 6; Figure 4). The seedling population TS4 has an allelic composition comprising several alleles shared with the northern populations, as well as the allele 293 for locus Pd32 and allele 243 for Ob11, both of which not found in the other populations (Table 6; Figure 5). The diversity of the seedling population at TS4, particularly at locus Pd32 makes it clear that these seedlings arose from more than one mother plant, consistent with local observations that there were previously up to three possible parent plants at this site. The genetic composition of the single planta at TS5 and TS6 suggests they could have arisen from dispersal events from nearby TS7 as all the alleles present in TS5 and TS6 are present in TS7(Table 6; Figures 1 and 5).

The population at TS7 contains a unique allele 267 at locus Ob11 and two unique alleles at locus Aacu07 (Table 6; Figure 1). Given the small size of this population, this allelic diversity suggests this population arose from multiple founder individuals or that there are/were other populations in the region. The greater diversity of TS7 combined with the nature of the environment in this region suggests the northern region may have been the center of diversity of this species. The UPGMA genetic clustering analysis suggests the lower diversity southern populations may have been more isolated and genetically less diverse with the exception of the TS4 population (Figure 5).

## 4 DISCUSSION

*Tahina spectabilis* was previously documented from a single location in northwestern Madagascar (Dransfield, Leroy, Metz, & Rakotoarinivo, 2008; Dransfield, Rakotoarinivo, et al., 2008), this area houses populations TS1, 2, and 3. The discovery of new locations of *T. spectabilis,* populations TS4, 5, 6, and 7, has considerably expanded its distribution range by orders of magnitude from the original IUCN Red List assessments, which gave both EOO and AOO as 4 km^2^ (Rakotoarinivo & Dransfield, 2012). The increase in the extent of the wild population, now EOO = 207 km^2^ and AOO = 24 km^2^, should reduce the extinction rate. However, when the IUCN red list criteria (IUCN, 2012) are reapplied for this species with the new data it still places its threat category as “Critically Endangered, CR” as there are fewer than 50 reproductively mature individuals throughout the whole wild population, thus criterion D corresponding to the CR category applies (IUCN, 2012). Further to this, conservation efforts to reintroduce the species within its natural range have approximately doubled these estimates but these plants will not reach maturity for many decades. The habitat suitability models have also identified considerable new areas where further wild populations may be discovered and new searches are ongoing and may result in more plants being located. A recent report by Humphreys et al. (2019) pointed out the large number of species thought to be extinct that have recently been rediscovered. While *T. spectabilis* was only relatively recently discovered, this study shows the value in targeted searches to discover new populations. In addition to the discovery of further locations of occurrence, this study also found that the largest known population (TS1) has approximately doubled its area between 2008 and 2016. However, it is important to notice that all known habitats are susceptible to degradation and decline in quality since the natural ecosystems in the area are generally threatened by the seasonal use of fire (Ackermann, 2003) and the potential effects of climate change such as severe flooding and increase of sedimentation due to erosion (Brown, Parks, Bethell, Johnson, & Mulligan, 2015).

Palms commonly have a “reverse J” population structure where seedling densities are considerably higher than that of juvenile and adults (Arneaud et al., 2017; Pettit & Dowe, 2003). Despite having a higher proportion of small sized plants, the population structure of *T. spectabilis* has a “pulsed” size structure (Figure 2). Our population stage structure and fertility was broadly similar to other studies of palms (e.g., Arneaud et al., 2017; Martínez‐Ramos, Ortiz‐Rodríguez, Piñero, Dirzo, & Sarukhán, 2010; Silva Matos, Freckleton, & Watkinson, 1999).Population level recruitment events seem to be relatively frequent in *T. spectabilis* and seed produced from these events appears to be usually viable despite being inbred. However, this has led to fluctuations in the size of the population that is capable of reproduction. Both reproductive success and offspring survival are required for population persistence (Bouzat, 2010; Brook, Tonkyn, O'Grady, & Frankham, 2002; Olmsted & Alvarez‐Buylla, 1995). Arneaud et al. (2017) found a high mortality in the young juvenile stage of some palm species and postulated this may be due to competition for resources and overcrowding.

We estimated that the lifespan of a *T. spectabilis* is on average 73 years before it flowers and dies, longer than initially predicted (Dransfield, Rakotoarinivo, et al., 2008), but this is expected to be highly variable and should be used as an approximate only. A broad age range has been found in other palm species (e.g., Martínez‐Ramos et al., 2010) and high variation among growth rates of individuals has also been previously reported for palm species (Jansen, Anten, Bongers, Martínez‐Ramos, & Zuidema, 2018). McPherson and Williams (1998) found that palms in fire prone environments can take at least two decades to attain an above‐ground trunk. Our estimates for *T. spectabilis* are consistent with these. In tropical regions with seasonally variable precipitation, flowering pulses in palms often occur according to precipitation patterns (Abrahamson, 1999). We do not know what triggers the initiation of flowering in *T. spectabilis* plants; however, the flowering events recorded during this study appear to coincide with the El Niño Southern Oscillation (ENSO) cycle and possibly the interaction of this and the Indian Ocean Dipole (IOD) (Fedorov & Philander, 2000; Gong et al., 2019).

The SDM models found that the species distribution is correlated with substantial seasonal rainfall (400–480 mm) during the wettest month (February) when *T.spectabilis* seedlings would be establishing. The predicted range area of *T. spectabilis* represents a particular ecological niche as it is located in the western climatic domain but also benefits from the marginal high moisture rate from the Sambirano region. The importance of low variation in temperature seasonality (43% of the distribution range) throughout the year emphasizes the role played by the heat in the growth physiology of palms by the absence of dormancy during the dry season (Tomlinson, 2006). Precipitation regime in the area is governed by the seasonal monsoon. The equatorial wind only reaches and has an influence on the climate of western Madagascar during the warm season. Summer rainfall is associated with heat and comes frequently with the intertropical convergence zone during which most of the annual precipitation is discharged in the area (Jury, 2016). A relatively high precipitation for a short or medium period, accompanied by flooding due to cyclone activity, sometimes results in the supply of a water table throughout the year despite the eight dry months. Groundwater table can be reached from 2 m underground (Upton, Dochartaigh, & BÉ, Monteleone M, Bellwood‐Howard I, 2018) in the predicted distribution range of *T. spectabilis*. If climate change leads to a higher rate of reproductive failure and seedling establishment then this would lead to reduced or slowed predicted population growth based on our current models.

We reported evidence of palm weevil in the fallen trunks of palms that had aborted their seed crop (Gardiner, Rabehevitra, & Rajaonilaza, 2017). Palm weevils are known to impact many palm species boring into old or damaged trunks (Azmi et al., 2017; Murphy & Briscoe, 1999; Rugman‐Jones, Hoddle, Hoddle, & Stouthamer, 2013). Their presence is concerning for this species though it is unknown if they caused the failure of the fruit set. While our population models indicate that reproductive failure could impact on *T. spectabilis* population growth, they also show that this seems unlikely to lead to its extinction. Previous demographic studies have shown that changes in individual palm growth rates and fecundity must be large and persistent to affect population growth rates (McPherson & Williams, 1998; Pinéro et al., 1984; Ratsirarson et al., 1996).

Extrinsic factors may have a major impact on the survival of the *T. spectabilis* population, as they can cause stochastic effects at the demographic level (Sodhi & Ehrlich, 2010) by exacerbating stress, and thus leading primarily to the death of vulnerable individuals. Many palms such as *T. spectabilis* inhabit fire prone environments and mature palms of many species are known to survive fires due to the distribution of meristematic tissue throughout the stem (Arneaud et al., 2017; Mandle, Ticktin, & Zuidema, 2015; McPherson & Williams, 1998). Previous studies have found that it is only the smallest size classes of seedlings in palms that are vulnerable to fire as the apical meristem is often well protected by leaf bases in juvenile stages (McPherson & Williams, 1998). Thus, our model accounts for the chance of fire leading to reduce survivorship of the seedling stages consistent with the levels reported in other studies (Arneaud et al., 2017; Mandle et al., 2015; McPherson & Williams, 1998). Our models suggest that fire alone is unlikely to lead to extinction of *T. spectabilis* populations. McPherson and Williams (1998) study also predicted that fire was insufficient to cause large changes in population growth rates and did not explain the rarity of their study species.

Grazing by herbivores such as cattle and goats has been documented to potentially impact on many palm species, particularly affecting the seedling stages, subsequently leading to reduced population growth (Mandle & Ticktin, 2012; Nazareno & dos Reis, 2014; Shapcott, Dowe, & Ford, 2009). The two largest *T. spectabilis* populations are closely associated with rocky outcrops. Some studies have found such habitats provide advantage for palms by increasing survivorship and reducing herbivory (Berry, Gorchov, Endress, & Stevens, 2008), and this may be the case for *T. spectabilis*. Many palm species are utilized by humans for a variety of purposes, such as the consumption of the palm heart and harvesting of palm leaves for thatch and a variety of other purposes (Gamba‐Trimiño et al., 2011; Gruca et al., 2016; Jansen et al., 2018; Martínez‐Ballesté et al., 2005; Navarro et al., 2011; Reis et al., 2000). Previous studies of palm demography have demonstrated that loss of palm leaves can reduce palm survival, growth and reproduction (Mandle & Ticktin, 2012; Martínez‐Ramos et al., 2010; Ratsirarson et al., 1996;). We found evidence that the leaves of *T. spectabilis* from TS7 are used locally for ceremonial mats, and this could impact viability and growth of individual plants in this very small population. Traditional harvesting may be sustainable in some instances, but there are varying estimates of sustainability from a variety of studies on a variety of harvested species (Jansen et al., 2018; Martínez‐Ballesté et al., 2005; Navarro et al., 2011). Thus for the population at TS7, it will be important to work with the local people to ensure that leaf harvesting is sustainable. Our population models that accounted for both reproductive failure and seedling mortality mimic the potential impacts of fire, grazing, harvesting, and insect attack and show that *T. spectabilis* is potentially tolerant to the combined effects of these. Mandle et al. (2015) also found the *Phoenix loureiroi* palms in India were resilient to low levels of fire, grazing, and harvesting.

The consumption of heart of palm has been documented as a widespread practice in Madagascar that potentially impacts on Critically Endangered palm species (Gruca et al., 2016; Shapcott et al., 2012). Given the long generation time of *T. spectabilis*, the critically small population sizes and the hapaxanthic reproductive lifecycle, this species is particularly vulnerable to impacts of heart of palm consumption as it kills the plants before they can reproduce (Gamba‐Trimiño et al., 2011; Shapcott et al., 2012).

Rare species are often found to have less genetic diversity than those species that are more abundant (Gitzendanner & Soltis, 2000; Leimu et al., 2006). While our study used a limited number of variable markers to assess genetic diversity, the low genetic variation found in *T. spectabilis* in this study is consistent with this expectation. Low genetic diversity has been found in other endangered Madagascan palm species (Gardiner, Rakotoarinivo, et al., 2017; Ratsirarson et al., 1996; Shapcott et al., 2012). However, the newly discovered population TS7 contained more genetic diversity than expected given the very small population size. Higher than expected genetic diversity has also been found in very small populations of the critically endangered Madagascan species *Voanioala gerardii* (Shapcott et al., 2012) and *Beccariophoenix madagascariensis* (Shapcott et al., 2007). Evidence from the genetic identity of TS2 indicates that it is a founder and arose by colonization from TS1. Likewise, TS5 and TS6 appear to have arisen from TS7 which indicates that the species can disperse seed at least a few km. Whereas, based on their allelic composition, TS3 and TS4 appear to have arisen from as yet undiscovered populations. Sezen, Chazdon, and Holsinger (2007) also found evidence of palm seed dispersal over similar distances and evidence of founder populations. The fruit of palms are an important food source for many animals, including lemurs (Arroyo‐Rodriguez, Aguirre, Benitez‐Malvido, & Mandujano, 2007; Dransfield, Uhl, et al., 2008; Gaiotto, Grattapaglia, & Vencovsky, 2003; Shapcott et al., 2007). Frugivorous birds and fruit bats are also known to be important for seed dispersal of palm species (Shapcott, 1998a, 1998b). Gaiotto et al. (2003) found that seeds of the heart of palm species *Euterpe edulis* had been dispersed by birds and become established up to 22 km away. Seed of *T. spectabilis* could potentially be dispersed across the landscape by fruit bats, parrots or lemurs which were observed or reported in the vicinity.

The main genetic consequences of fragmentation include reduced genetic diversity and increased population differentiation (Leimu, Vergeer, Angeloni, & Ouborg, 2010). Habitat fragmentation is expected to lead to random patterns of genetic differentiation and allele loss due to drift (Nistelberger, Coates, Llorens, Yates, & Byrne, 2015). Populations on the edge of the geographical species range are predicted to be less genetically diverse than those in the species center (Lawton, 1993). Cibrián‐Jaramillo et al. (2009) found evidence of a pattern of palm expansion via stepping stone founder populations radiating out from around regional clusters. Our results appear to show a pattern of radiation by founder populations from a center of diversity associated with the northern region. *Tahina spectabilis* displays geographic clustering similar to the genetic patterns found in *Lemurophoenix halleuxii* (Shapcott et al., 2012) and *Beccariophoenix madagascariensis* (Shapcott et al., 2007).

Heywood (2019) identifies the urgent need for action to prevent further plant extinctions. Conservation in the form of reintroduction and translocation carries elements of risk, and there are relatively few reported cases of successful establishment of translocation species (Godefroid et al., 2011; Silcock et al., 2019; Weeks et al., 2011). *Tahina spectabilis* seeds were wild harvested and distributed nationally and internationally as part of a conservation program initiated shortly after the species' discovery (Gardiner, Rabehevitra, Letsara, et al., 2017; Gardiner, Rabehevitra, & Rajaonilaza, 2017). The maintenance of genetic diversity and identity of regions must be considered carefully, both when sourcing of germplasm and introducing new material (Byrne et al., 2011; Mckay, Christian, Harrison, & Rice, 2005; Weeks et al., 2011). The planted populations of *T. spectablis* were sourced from the closest known source but they have very low genetic diversity as the seed arose from only one or two parent plants. We report that there are now five new planted populations that have been established in Madagascar within the adjacent area previously modelled as suitable for *T. spectabilis*. These planted populations have the potential to contribute to expanding *T. spectabilis* distribution extent to the south of its current known range including into a protected area. These new populations have the potential to contribute to considerable population increase in around 50–100 years as the trees mature and produce seed and at that time they will alleviate pressure on wild populations for commercial seed supplies for horticulture.

This study has found that while the total number of *T. spectabilis* plants has increased and the Area of occupancy (AOO) and Extent of occurrence has expanded (EOO) since its discovery the number of plants of a size capable of reproduction has declined since 2008 and remains at a critically small size thus the species remains Critically Endangered according to the IUCN criteria (D). Our modelling shows that if the current known populations remain protected by local customs and new conservation practices (Gardiner, Rabehevitra, Letsara, et al., 2017; Gardiner, Rabehevitra, & Rajaonilaza, 2017), *T. spectabilis* populations are expected to maintain themselves and eventually expand and we expect to see a recovery in the next 100 years. If new populations are discovered as a result of recent searches this will assist to ensure the species long‐term viability if new discoveries are accompanied by local conservation security actions. However, the species appears sensitive to seasonal rainfall with this predicting its distribution and potentially its reproductive events and thus may be impacted by climate change in the longer term.

## CONFLICT OF INTEREST

There are no competing interests.

## AUTHOR CONTRIBUTIONS

AS lead the collection of field data in 2008 and 20,016, analyzed the final genetics, and demographic data undertook demographic modelling, supervised student HJ and was principal manuscript writer. HJ undertook initial genetic and demographic analysis and contributed to writing especially methods.LS undertook second genetic analysis and contributed to PVA model methods and critical review and editing manuscript. YS contributed to developing demographic models and PVA and critical review of the manuscript. LG obtained funding and lead the 2016 field trip conceptual design as well as contributing to critical manuscript writing and revision. DR organized local field logistics and contributed to the field work in the 2016 trip as well as developing conservation priorities for the species and contributed to manuscript writing. RL participated in the 2016 field trip and liased with local authorities and Botanic Gardens to obtain permits as well as contributing to the critical manuscript revision. SC was responsible for the Kew logistics team and both grant and permit and contributed to critical manuscript editing and who has been key in the establishing of the species conservation program. JD provided ongoing taxonomic, observational and logistical advice and was responsible for making the original connection to Xavier Metz who discovered the species, assisted with the establishment of the species conservation program and C de Foucault who has conserved the species in planted population as well as contributing to the critical review of interpretation of results and manuscript. WB has supported this project in his lead role at RBGKEW from its inception and had contributed critical review of all versions of manuscripts and data interpretation from the original Honours thesis to this final paper. MR assisted with permits and logistical support for the 2008 trip, developed SDM models and made a major contribution to the manuscript writing and editing. All listed authors have read and approved the final submitted manuscript.

## Data Availability

We used previously published microsatellite markers and cited the references for their source. Since the species is critically endangered the precise locations have not been given for all sites however the main natural populations have Herbarium voucher specimens that are lodged with the Royal Botanic Gardens Kew and hence accessible via their databases. The raw data used for analyses will be lodged with the University of the Sunshine Coast Research Data Repository and made available upon request after publication.

## References

[ece36137-bib-0001] Abrahamson, W. G. (1999). Episodic Reproduction in Two Fire‐Prone Palms, *Serenoa repens* and *Sabal etonia* (Palmae). Ecology, 80, 100–115.

[ece36137-bib-0002] Ackermann, K. (2003). The role of dry forests in Madagascar as a safety net in the rural livelihood system. The International Conference on Rural Livelihoods, Forests and Biodiversity. Bonn, Germany.

[ece36137-bib-0003] Akçakaya, H. R. (2005). RAMAS GIS: Linking spatial data with population viability analysis version 5. Setauket, NY: Applied Biomathematics.

[ece36137-bib-0004] Akçakaya, H. R. , Burgman, M. A. , & Ginzbur, L. R. (1999). Applied population ecology. Principles and computer exercises using RAMAS Ecolab (2nd edn). Sunderland, MA: Sinauer Associates Inc.

[ece36137-bib-0005] Arneaud, L. L. , Farrell, A. D. , & Oatham, M. P. (2017). Marked reproductive plasticity in response to contrasting fire regimes in a neotropical palm. Tropical Ecology, 58(4), 693–703.

[ece36137-bib-0006] Arroyo‐Rodriguez, V. , Aguirre, A. , Benitez‐Malvido, J. , & Mandujano, S. (2007). Impact of rain forest fragmentation on the population size of a structurally important palm species: *Astrocaryum mexicanum* at Los Tuxtlas. Mexico Biological Conservation, 138, 198–206. 10.1016/j.biocon.2007.04.016

[ece36137-bib-0007] Azmi, A. W. , Lian, C. J. , Zakeri, H. A. , Yusuf, N. , Omar, W. B. W. , Wai, Y. K. , … Husasin, M. H. (2017). The Red Palm Weevil, *Rhynchophorus ferrugineus*: Current issues and challenges in Malaysia. Oil Palm Bulletin, 74, 17–24.

[ece36137-bib-0008] Baker, W. J. , Savolainen, V. , Asmussen‐Lange, C. B. , Chase, M. W. , Dransfield, J. , Forest, F. , … Wilkinson, M. (2009). Complete generic‐level phylogenetic analyses of palms (Arecaceae) with comparisons of supertree and supermatrix approaches. Systematic Biology, 58, 240–256. 10.1093/sysbio/syp021 20525581

[ece36137-bib-0009] Berry, E. J. , Gorchov, D. L. , Endress, B. A. , & Stevens, M. H. H. (2008). Source‐sink dynamics within a plant population: The impact of substrate and herbivory on palm demography. Population Ecology, 50, 63–77. 10.1007/s10144-007-0067-z

[ece36137-bib-0010] Besairie, H. (1964). Carte géologique de Madagascar à l'échelle de 1/1 000000. Tananarive, Madagascar: Service géologique de Madagascar.

[ece36137-bib-0011] Billotte, N. , Marseillac, N. , Brottier, P. , Noyer, J. L. , Jacquemoud‐Collet, J. P. , Moreau, C. , … Risterucci, A. M. (2004). Nuclear microsatellite markers for the date palm (*Phoenix dactylifera* L.): Characterization and utility across the genus *Phoenix* and in other palm genera. Molecular Ecology Notes, 4, 256–258. 10.1111/j.1471-8286.2004.00634.x

[ece36137-bib-0012] Billotte, N. , Risterucci, A. M. , Barcelos, E. , Noyer, J. L. , Amblard, P. , & Baurens, F. C. (2001). Development, characterization and cross‐taxa utility of oil palm (*Elaeis guineensis* Jacq) microsatellite markers. Genome, 44, 413–425. 11444700

[ece36137-bib-0013] Bouzat, J. (2010). Conservation genetics of population bottlenecks: The role of chance, selection, and history. Conservation Genetics, 11, 463–478. 10.1007/s10592-010-0049-0

[ece36137-bib-0014] Brook, B. W. , Tonkyn, D. W. , O'Grady, J. J. , & Frankham, R. (2002). Contribution of inbreeding to extinction risk in threatened species. Conservation Ecology, 6(1), 1–16. 10.5751/ES-00387-060116

[ece36137-bib-0015] Brown, K. A. , Parks, K. E. , Bethell, C. A. , Johnson, S. E. , & Mulligan, M. (2015). Predicting plant diversity patterns in Madagascar: Understanding the effects of climate and land cover change in a biodiversity hotspot. PLoS ONE, 10(4), e0122721 10.1371/journal.pone.0122721 25856241 PMC4391717

[ece36137-bib-0016] Byrne, M. , Stone, L. , & Millar, M. (2011). Assessing genetic risk in revegetation. Journal of Applied Ecology, 48, 1365–1373. 10.1111/j.1365-2664.2011.02045.x

[ece36137-bib-0017] Cibrián‐Jaramillo, A. , Bacon, C. D. , Garwon, N. C. , Bateman, R. M. , Thomas, M. M. , Russell, S. , … DeSalle, R. (2009). Population genetics of the understory fishtail palm *Chamaedorea ernesti‐augusti* in Belize: High genetic connectivity with local differentiation. BMC Genetics, 10, 65 10.1186/1471-2156-10-65 19818141 PMC2770526

[ece36137-bib-0018] Clarke, K. , & Gorley, R. (2001). Plymouth routines in multivariate ecological research. Plymouth, UK: PRIMER‐E Ltd.

[ece36137-bib-0019] Dransfield, J. , & Beentje, H. (1995). The palms of Madagascar. London, UK: Royal Botanic Gardens Kew/International Palm Society, Kew.

[ece36137-bib-0020] Dransfield, J. , Leroy, B. , Metz, X. , & Rakotoarinivo, M. (2008). Tahina – A new palm genus from Madagascar. Palms, 52, 31–39.

[ece36137-bib-0021] Dransfield, J. , & Rakotoarinivo, M. (2011). The biology of island floras: The biogeography of Madagascar palms. Cambridge, UK: Cambridge University Press 10.1017/CBO9780511844270.008

[ece36137-bib-0022] Dransfield, J. , Rakotoarinivo, M. , Baker, W. J. , Bayton, R. P. , Fisher, J. B. , Horn, J. W. , … Metz, X. (2008). A new Coryphoid palm genus from Madagascar. Botanical Journal of the Linnean Society, 156(1), 79–91. 10.1111/j.1095-8339.2007.00742.x

[ece36137-bib-0023] Dransfield, J. , Uhl, N. W. , Asmussen, C. B. , Baker, W. J. , Harley, M. M. , & Lewis, C. E. (2008). Genera Palmarum – The evolution and classification of palms. Richmond, UK: Royal Botanic Gardens, Kew.

[ece36137-bib-0024] Fedorov, A. V. , & Philander, S. G. (2000). Is El Niño changing? Science, 288, 1197–2002. 10.1126/science.288.5473.1997 10856205

[ece36137-bib-0025] Gaiotto, F. A. , Grattapaglia, D. , & Vencovsky, R. (2003). Genetic structure, mating system and long‐distance gene flow in heart of palm (*Euterpe edulis* Mart.). Journal of Heredity, 94, 399–406. 10.1093/jhered/esg087 14557393

[ece36137-bib-0026] Gamba‐Trimiño, C. , Bernal, R. , & Bittner, J. (2011). Demography of the clonal palm *Prestoea acuminata* in the Colombian Andes: Sustainable household extraction of palm hearts. Tropical Conservation Science, 4(4), 386–404.

[ece36137-bib-0027] Gardiner, L. M. , Rabehevitra, D. , Letsara, R. , & Shapcott, A. (2017). *Tahina spectabilis*: An exciting new discovery in Madagascar ten years on. Palms, 61(2), 69–82.

[ece36137-bib-0028] Gardiner, L. M. , Rabehevitra, D. , & Rajaonilaza, T. (2017). Madagascar plant conservation management plan *– Tahina spectabilis*. Richmond, UK: Royal Botanic Gardens, Kew. Published Online.

[ece36137-bib-0029] Gardiner, L. M. , Rakotoarinivo, M. , Rajaovelona, L. R. , & Clubbe, C. (2017). Population genetics data help to guide the conservation of palm species with small population sizes and fragmented habitats in Madagascar. PeerJ, 5, e3248 10.7717/peerj.3248 28480141 PMC5419215

[ece36137-bib-0030] Gautier, L. , & Goodman, S. M. (2003). Introduction to the flora of Madagascar In Goodman S. M., & Benstead J. P. (Eds.), The natural history of Madagascar (pp. 229–250). Chicago, IL: University of Chicago Press.

[ece36137-bib-0031] Gitzendanner, M. A. , & Soltis, P. (2000). Patterns of genetic variation in rare and widespread plant congeners. American Journal of Botany, 87, 783–792. 10.2307/2656886 10860909

[ece36137-bib-0032] Godefroid, S. , Piazza, C. , Rossi, G. , Buord, S. , Stevens, A.‐D. , Aguraiuja, R. , … Vanderborght, T. (2011). How successful are plant species reintroductions? Biological Conservation, 144, 672–682. 10.1016/j.biocon.2010.10.003

[ece36137-bib-0033] Gong, H. , Zhou, W. , Chen, W. , Wang, L. , Leung, M.‐T. , Cheung, P.‐Y. , & Zhang, Y. (2019). Modulation of the southern Indian Ocean dipole on the impact of El Niño‐Southern Oscillation on Australian summer rainfall. International Journal of Climatology, 39, 2484–2490. 10.1002/joc.5941

[ece36137-bib-0034] Govaerts, R. , Dransfield, J. , Zona, S. , Hodel, D. R. , & Henderson, A. (2019). World checklist of Arecaceae. Facilitated by the Royal Botanic Gardens, Kew, Richmond, UK Published on the Internet; Retrieved Oct 2019 from http://wcsp.science.kew.org/

[ece36137-bib-0035] Gruca, M. , Blanch‐Overgaard, A. , Dransfield, J. , & Balslev, H. (2016). Medicinal palms (Arecaceae) in Madagascar—undocumented or underutilized? Botanical Journal of the Linnean Society, 182, 517–525. 10.1111/boj.12422

[ece36137-bib-0036] Heywood, V. H. (2019). Conserving plants within and beyond protected areas – still problematic and future uncertain. Plant Diversity, 41, 36–49. 10.1016/j.pld.2018.10.001 31193163 PMC6520483

[ece36137-bib-0037] Hijmans, R. J. , Cameron, S. E. , Parra, J. L. , Jones, P. G. , & Jarvis, A. (2005). Very high resolution interpolated climate surfaces for global land areas. International Journal of Climate., 25, 1965–1978. 10.1002/joc.1276

[ece36137-bib-0038] Hooftman, D. , & Diemer, M. (2002). Effects of small habitat size and isolation on the population structure of common wetland species. Plant Biology, 4, 720–728. 10.1055/s-2002-37400

[ece36137-bib-0039] Humphreys, A. M. , Govaerts, R. , Fininski, S. Z. , Lughadha, E. N. , & Vorontsova, M. S. (2019). Global dataset shows geography and life form predict modern plant extinction and rediscovery. Nature Ecology & Evolution, 3, 1043–1047. 10.1038/s41559-019-0906-2 31182811

[ece36137-bib-0040] IUCN . (2012). UCN Red List categories and criteria, version 3.1 (2nd ed., p. 32). Gland and Cambridge: IUCN.

[ece36137-bib-0041] IUCN Standards and Petitions Subcommittee . (2014). Guidelines for using the IUCN red list categories and criteria. Version 11. Prepared by the Standards and Petitions Subcommittee.

[ece36137-bib-0043] James, H. J. (2010). Conservation genetics of *Tahina spectabilis*, newly discovered palm from Madagascar. Honours Thesis, University of the Sunshine Coast, Queensland, Australia.

[ece36137-bib-0044] Jansen, M. , Anten, N. P. R. , Bongers, F. , Martínez‐Ramos, M. , & Zuidema, P. A. (2018). Towards smarter harvesting from natural palm populations by sparing the individuals that contribute most to population growth or productivity. Journal of Applied Ecology, 55, 1682–1691. 10.1111/1365-2664.13100

[ece36137-bib-0045] Jiang, Z. , Huete, A. R. , Chen, J. , Chen, Y. , Li, J. , Yan, G. , & Zhang, X. (2006). Analysis of NDVI and scaled difference vegetation index retrievals of vegetation fraction. Remote Sensing of Environment, 101(3), 366–378. 10.1016/j.rse.2006.01.003

[ece36137-bib-0046] Jury, M. R. (2003). The climate of Madagascar In Goodman S. M., & Benstead J. P. (Eds), The natural history of Madagascar (pp. 75–87). Chicago, IL: The University of Chicago Press.

[ece36137-bib-0047] Jury, M. R. (2016). Summer climate of Madagascar and monsoon pulsing of its vortex. Meteorology and Atmospheric Physics, 128(1), 117 10.1007/s00703-015-0401-5

[ece36137-bib-0048] Kull, C. A. (2002). Empowering pyromaniacs in Madagascar; ideology and legitimacy in community‐based natural resource management. Development and Change, 33, 57–78. 10.1111/1467-7660.00240

[ece36137-bib-0049] Lawton, J. (1993). Range, population abundance and conservation. Trends in Ecology and Evolution, 8, 409–413. 10.1016/0169-5347(93)90043-O 21236213

[ece36137-bib-0050] Leimu, R. , Mutiikainen, P. , Koricheva, J. , & Fischer, M. (2006). How general are positive relationships between plant population size, fitness and genetic variation? Journal of Ecology, 94, 942–952. 10.1111/j.1365-2745.2006.01150.x

[ece36137-bib-0051] Leimu, R. , Vergeer, P. , Angeloni, F. , & Ouborg, N. J. (2010). Habitat fragmentation, climate change, and inbreeding in plants. Annals of the New York Academy of Science, 1195, 84–98. 10.1111/j.1749-6632.2010.05450.x 20536818

[ece36137-bib-0052] Mandle, L. , & Ticktin, T. (2012). Interactions among fire, grazing, harvest and abiotic conditions shape palm demographic responses to disturbance. Journal of Ecology, 100, 997–1008. 10.1111/j.1365-2745.2012.01982.x

[ece36137-bib-0053] Mandle, L. , Ticktin, T. , & Zuidema, P. A. (2015). Resilience of palm populations to disturbance is determined by interactive effects of fire, herbivory and harvest. Journal of Ecology, 103, 1032–1043. 10.1111/1365-2745.12420

[ece36137-bib-0054] Martínez‐Ballesté, A. , Martorell, C. , Martínez‐Ramos, M. , & Caballero, J. (2005). Applying retrospective demographic models to assess sustainable use the Maya management of xa'an palms. Ecology and Society, 10, 17.

[ece36137-bib-0055] Martínez‐Ramos, M. , Ortiz‐Rodríguez, I. A. , Piñero, D. , Dirzo, R. , & Sarukhán, J. (2010). Anthropogenic disturbances jeopardize biodiversity conservation within tropical rainforest reserves. Proceedings of the National Academy of Sciences of the United States of America, 113(19), 5323–5328. 10.1073/pnas.1602893113 PMC4868451 27071122

[ece36137-bib-0056] McKay, J. K. , Christian, C. E. , Harrison, S. , & Rice, K. J. (2005). How local is local? – A review of practical and conceptual issues in the genetics of restoration. Restoration Ecology, 13, 432–440. 10.1111/j.1526-100X.2005.00058.x

[ece36137-bib-0057] McPherson, K. , & Williams, K. (1998). Fire resistance of cabbage palms (*Sabal palmetto*) in the southeastern USA. Forest Ecology and Management, 109, 197–207. 10.1016/S0378-1127(98)00243-6

[ece36137-bib-0058] Melito, M. O. , Faria, J. C. , Amorim, A. M. , & Cazetta, E. (2014). Demographic structure of a threatened palm (*Euterpe edulis* Mart.) in a fragmented landscape of Atlantic Forest in northeastern Brazil. Acta Botanica Brasilica, 28(2), 249–258.

[ece36137-bib-0059] Meyers, N. , Mittermeier, R. A. , Mittermeier, C. G. , da Fonseca, G. A. B. , & Kents, J. (2000). Biodiversity hot spots for conservation priorities. Nature, 403, 853–858. 10.1038/35002501 10706275

[ece36137-bib-0060] Moat, J. , & Smith, P. (2007). Atlas of the vegetation of Madagascar. Royal Botanic Gardens Kew, UK, 124 p.

[ece36137-bib-0061] Montufar, R. , Mariac, C. , Pham, J. L. , & Pintaud, J. C. (2007). Isolation of 23 polymorphic microsatellite loci in the Neotropical palm *Oenocarpus bataua* Martius (Arecaceae). Molecular Ecology Notes, 7, 75–78. 10.1111/j.1471-8286.2006.01532.x

[ece36137-bib-0062] Murphy, S. T. , & Briscoe, B. R. (1999). The red palm weevil as an alien invasive: Biology and the prospects for biological control as a component of IPM. Biocontrol News and Information, 20, 35–46.

[ece36137-bib-0063] Navarro, J. , Galeano, G. , & Bernal, R. (2011). Impact of leaf harvest on populations of *Lepidocaryum tenue*, an Amazonian understory palm used for thatching. Tropical Conservation Science, 4(1), 25–38.

[ece36137-bib-0064] Nazareno, A. G. & dos Reis, M. S. (2014). At risk of population decline? An ecological and genetic approach to the threatened palm species *Butia eriospatha* (Arecaceae) of Southern Brazil. Journal of Heredity, 105(1):120–129. 10.1093/jhered/est065 24078681

[ece36137-bib-0065] Nistelberger, H. M. , Coates, D. J. , Llorens, T. M. , Yates, C. J. , & Byrne, M. (2015). A cryptic genetic boundary in remnant populations of a long‐lived, bird‐pollinated shrub *Banksia sphaerocarpa* var. *caesia* (Proteaceae). Biological Journal of the Linnean Society, 115, 241–255.

[ece36137-bib-0066] Nucci, S. , Azevedo‐Filho, J. , Colombo, C. , Priolli, R. , Coelho, R. , Mata, T. , & Zucchi, M. (2008). Development and characterization of microsatellites markers from the macaw. Molecular Ecology, 8, 224–226. 10.1111/j.1471-8286.2007.01932.x 21585762

[ece36137-bib-0067] Olmsted, I. , & Alvarez‐Buylla, E. (1995). Sustainable harvesting of tropical trees: Demography and matrix models of two palm species in Mexico. Ecological Applications, 5, 484–500. 10.2307/1942038

[ece36137-bib-0069] Peakall, R. , & Smouse, P. E. (2006). GENALEX 6: Genetic analysis in Excel. Population genetic software for teaching and research. Molecular Ecology Notes, 6, 288–295. 10.1111/j.1471-8286.2005.01155.x PMC3463245 22820204

[ece36137-bib-0070] Pettit, N. E. , & Dowe, J. L. (2003). Distribution and population structure of the vulnerable riparian palm *Livistona lanuginosa* A. N. Rodd (Arecaceae) in the Burdekin River catchment, north Queensland. Pacific Conservation Biology, 9, 207–214.

[ece36137-bib-0071] Pfab, M. F. , & Witkowski, E. T. F. (1997). Use of Geographical Information Systems in the search for additional populations, or sites suitable for re‐establishment, of the endangered Northern Province endemic *Euphorbia clivicola* . South African Journal of Botany, 63, 351–355.

[ece36137-bib-0072] Phillips, S. J. , Anderson, R. P. , & Schapire, R. E. (2006). Maximum entropy modeling of species geographic distributions. Ecological Modelling, 190, 231–259. 10.1016/j.ecolmodel.2005.03.026

[ece36137-bib-0073] Pinéro, D. , Martinez‐Ramos, M. , & Sarukhan, J. (1984). A population model of *Astrocaryum Mexicanum* and a sensitivity analysis of its finite rate of increase. Journal of Ecology, 72, 977–991. 10.2307/2259545

[ece36137-bib-0074] Rakotoarinivo, M. , & Dransfield, J. (2012). *Tahina spectabilis*. The IUCN Red List of Threatened Species 2012: e.T195893A2430024. 10.2305/IUCN.UK.2012.RLTS.T195893A2430024.en

[ece36137-bib-0075] Rakotoarinivo, M. , Dransfield, J. , Bachman, S. P. , Moat, J. , & Baker, W. J. (2014). Comprehensive red list assessment reveals exceptionally high extinction risk to Madagascar palms. PLoS ONE, 9(7), e103684 10.1371/journal.pone.0103684 25075612 PMC4116232

[ece36137-bib-0076] Ratsirarson, J. , Silander, J. A. Jr , & Richard, A. F. (1996). Conservation and management of a threatened Madagascar palm species, *Neodypsis decaryi*, Jumelle. Conservation Biology, 10, 40–52. 10.1046/j.1523-1739.1996.10010040.x

[ece36137-bib-0077] Reis, M. S. , Fantini, A. C. , Nodari, R. O. , Reis, A. , Guerra, M. P. , & Mantovani, A. (2000). Management and conservation of natural populations in Atlantic rain forest: The case study of palm heart (*Euterpe edulis* Martius). Biotropica, 32(4b), 894–902.

[ece36137-bib-0078] Rugman‐Jones, P. F. , Hoddle, C. D. , Hoddle, M. S. , & Stouthamer, R. (2013). The lesser of two weevils: Molecular‐genetics of pest palm weevil populations confirm *Rhynchophorus vulneratus* (Panzer 1798) as a valid species distinct from *R. ferrugineus* (Olivier 1790), and reveal the global extent of both. PLoS ONE, 8(10), e78379 10.1371/journal.pone.0078379 24143263 PMC3797061

[ece36137-bib-0079] Sezen, U. U. , Chazdon, R. L. , & Holsinger, K. E. (2007). Multigenerational genetic analysis of tropical secondary regeneration in a canopy palm. Ecology, 88, 3065–3075. 10.1890/06-1084.1 18229841

[ece36137-bib-0080] Shapcott, A. (1998a). The patterns of genetic diversity in *Carpentaria acuminata* (Arecaceae), and rainforest history in northern Australia. Molecular Ecology, 7, 833–847.

[ece36137-bib-0081] Shapcott, A. (1998b). The genetics of *Ptychosperma bleeseri* a rare palm from the Northern Territory. Australia. Biological Conservation, 85, 203–209. 10.1016/S0006-3207(97)00147-X

[ece36137-bib-0082] Shapcott, A. , Dowe, J. , & Ford, H. (2009). Low genetic diversity and recovery implications of the vulnerable Bankouale Palm *Livistona carinensis* (Arecaceae), from North‐eastern Africa and the Southern Arabian Peninsula. Conservation Genetics, 10, 317–327.

[ece36137-bib-0083] Shapcott, A. , Lamont, R. W. , Conroy, G. , James, H. E. , & Shimizu‐Kimura, Y. (2017). Genetics and species distribution modelling of *Solanum johnsonianum* (Solanaceae*)* reveal impacts of brigalow land clearing on this endemic species. Conservation Genetics, 18(6), 1331–1346. 10.1007/s10592-017-0983-1

[ece36137-bib-0084] Shapcott, A. , & Powell, M. (2011). Demographic structure, genetic diversity and habitat distribution of the endangered, Australian rainforest tree *Macadamia jansenii* help facilitate a reintroduction program. Australian Journal of Botany, 59, 215–225.

[ece36137-bib-0085] Shapcott, A. , Quinn, J. , Rakotoarinivo, M. , & Dransfield, J. (2012). Contrasting patterns of Genetic diversity between two endangered palms with overlapping distributions, *Voanioala gerardii* (Arecoideae) and *Lemurophoenix halleuxii* (Arecoideae), from North‐east Madagascar. Conservation Genetics, 13, 1393–1408. 10.1007/s10592-012-0382-6

[ece36137-bib-0086] Shapcott, A. , Rakotoarinivo, M. , Smith, R. , Lysakova, G. , Fay, M. , & Dransfield, J. (2007). Can we bring Madagascar's critically endangered palms back from the brink? Genetics, ecology and conservation of the critically endangered palm *Beccariophoenix madagascariensis* . Botanical Journal of the Linnean Society, 154, 589–608. 10.1111/j.1095-8339.2007.00676.x

[ece36137-bib-0087] Silcock, J. L. , Simmons, C. L. , Monks, L. , Dillon, R. , Reiter, N. , Jusaitis, M. , … Coates, D. J. (2019). Threatened plant translocation in Australia: A review. Biological Conservation, 236, 211–222. 10.1016/j.biocon.2019.05.002

[ece36137-bib-0088] Silva Matos, D. M. , Freckleton, R. P. , & Watkinson, A. R. (1999). The role of density dependence in the population dynamics of a tropical palm. Ecology, 80(8), 2635–2650. 10.1890/0012-9658(1999)080[2635:TRODDI]2.0.CO;2

[ece36137-bib-0089] Simmons, C. L. , Auld, T. D. , Hutton, I. , Baker, W. J. , & Shapcott, A. (2012). Will climate change, genetic and demographic variation or rat predation pose the greatest risk of persistence of an altitudinally disturbed island endemic? Biology, 1, 736–765. 24832517 10.3390/biology1030736 PMC4009823

[ece36137-bib-0090] Sodhi, N. S. , & Ehrlich, P. R. (2010). Conservation biology for all. Oxford, UK: Oxford University Press 10.1093/acprof:oso/9780199554232.001.0001

[ece36137-bib-0091] Thode, V. A. , Silva‐Arias, G. A. , Turchetto, C. , Segatto, A. L. A. , Mäder, G. , Bonatto, S. L. , & De Freitas, L. B. (2014). Genetic diversity and ecological niche modelling of the restricted *Recordia reitzii* (Verbenaceae) from southern Brazilian Atlantic forest. Botanical Journal of the Linnean Society, 176, 332–348.

[ece36137-bib-0092] Tomlinson, P. B. (2006). The uniqueness of palms. Botanical Journal of the Linnean Society, 151, 5–14. 10.1111/j.1095-8339.2006.00520.x

[ece36137-bib-0093] Upton, K. , Ó Dochartaigh, B. É. , Monteleone, M. , & Bellwood‐Howard, I. (2018). Africa Groundwater Atlas: Hydrogeology of Madagascar. British Geological Survey. Retrieved from http://earthwise.bgs.ac.uk/index.php/Hydrogeology_of_Madagascar. Accessed October 31, 2019.

[ece36137-bib-0094] US Geological Survey (2015). Global 30 arc‐second elevation. Retrieved from https://lta.cr.usgs.gov/gtopo30

[ece36137-bib-0095] Weeks, A. R. , Sgro, C. M. , Young, A. G. , Frankham, R. , Mitchell, M. J. , Miller, K. A. , … Hoffman, A. A. (2011). Assessing the benefits and risks of translocations in changing environments: A genetic perspective. Evolutionary Applications, 4, 705–724. 10.1111/j.1752-4571.2011.00192.x PMC3265713 22287981

